# Aquaporin 3 facilitates chemoresistance in gastric cancer cells to cisplatin *via* autophagy

**DOI:** 10.1038/cddiscovery.2016.87

**Published:** 2016-11-14

**Authors:** Xuqiang Dong, Yao Wang, Yangchun Zhou, Jianfei Wen, Shoulin Wang, Lizong Shen

**Affiliations:** 1 Division of Gastrointestinal Surgery, Department of General Surgery, First Affiliated Hospital, Nanjing Medical University, Nanjing 210029, China; 2 Department of General Surgery, Sir Run Run Hospital, Nanjing Medical University, Nanjing 211166, China; 3 School of Public Health, Nanjing Medical University, Nanjing 211166, China

## Abstract

Cisplatin (cDDP) remains one of the first-line chemotherapeutic agents for gastric cancer (GC) treatment, and resistance to cDDP is the major limitation in its clinical application. Mechanisms of cDDP resistance have been shown to be varied and complicated. Aquaporin 3 (AQP3) has been demonstrated to be overexpressed in GC tissues and is thought to be involved in GC carcinogenesis and progression. However, the role of AQP3 in chemosensitivity of GC to cytotoxic agents remains unknown. In this study, we show that AQP3 overexpression induced resistance to cDDP in AGS cells (*P*<0.05), and AQP3 knockdown increased the chemosensitivity in MGC803 and SGC7901 cells (*P*<0.05). Moreover, cDDP treatment enhanced AQP3 expression in MGC803, SGC7901 and AGS cells. AQP3 overexpression promoted the conversion of LC3-I to LC3-II in AGS cells, whereas AQP3 knockdown inhibited this conversion in MGC803 and SGC7901 cells. AQP3 upregulation increased Atg5 and Beclin-1 expression, and inhibited P62 expression in AGS cells, whereas AQP3 knockdown showed the opposite results in MGC803 and SGC7901 cells. Chloroquine (CQ), an autophagy inhibitor, enhanced the cytotoxicity of cDDP in GC cells, and CQ reversed the chemoresistance to cDDP caused by AQP3 overexpression in GC cells. Together, our data demonstrate that AQP3 facilitates cisplatin resistance in gastric cancer cells *via* autophagy, and suggest that the development of AQP3-based tumor therapeutics could play a key role in future GC treatment strategies.

## Introduction

Gastric carcinoma (GC) remains one of the most common and lethal malignancies worldwide, especially in China.^1^ Despite the development of new therapeutic strategies in recent decades, radical surgery and platinum-based chemotherapy are still the standard treatments for GC, and cisplatin (*cis*-diamminedichloroplatinum (cDDP)) is one of the first-line chemotherapeutic agents for GC.^2^ Cisplatin exerts its cytotoxic effect predominantly by forming intrastrand crosslinks in DNA that block transcription and DNA replication, resulting in cell apoptosis.^3^ However, resistance to cDDP in GC is increasing, leading to a major limitation in its clinical application.^4^ Mechanisms of cDDP resistance are complicated, including decreased drug uptake, increased drug efflux, increased DNA damage repair, alterations in apoptotic signaling pathways^5^ and induced autophagy.^6^ Understanding the mechanism of cDDP resistance is crucial for GC therapy.

Aquaporins (AQPs) are a family of small, integral membrane proteins that facilitate water transport across the plasma membranes of cells in response to osmotic gradients, and in some cases AQPs can transport glycerol (‘aquaglyceroporins’).^7^ AQP3 is a member of the aquaglyceroporin family that has been recently been found to transport H_2_O_2_.^8,9^ Mounting evidence further implicates AQPs in promoting cancer cell migration and proliferation, adding AQPs to an expanding list of effectors in tumor biology.^10^ Our previous study demonstrated that AQP3 is overexpressed in GC tissues, that its expression is associated with histological classification, lymph node metastasis and lymphovascular invasion^7,11^ and that its upregulation promotes the invasion and metastasis of GC cells *via* promoting the epithelial–mesenchymal transition (EMT) and the stem-like properties of gastric cancer cells.^12,13^

Although AQP3 overexpression has been demonstrated to contribute to chemoresistance in melanoma to arsenite,^14^ little is known about its role in chemosensitivity of GC to cytotoxic agents. In this study, we showed for the first time that AQP3 mediates chemoresistance in gastric cancer cells to cisplatin, enhances autophagy of gastric cancer cells and the lysosome inhibitor chloroquine reverses the chemoresistance induced by AQP3. Collectively, our results demonstrate that AQP3 facilitates cisplatin resistance in gastric cancer cells *via* autophagy.

## Results

### AQP3 mediates chemoresistance in GC cells to cDDP

**Figure 1:**
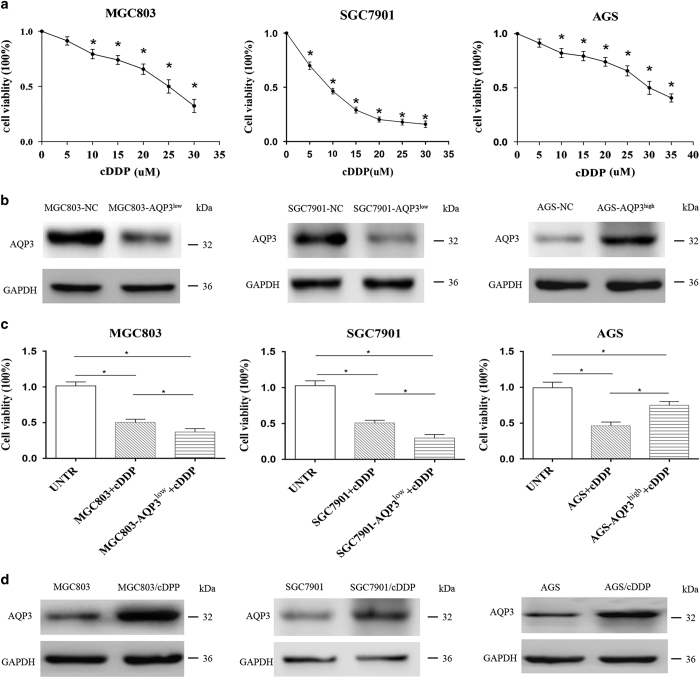
Aquaporin 3 (AQP3) mediates chemoresistance in gastric cancer cells to cisplatin (cDDP). (**a**) cDDP exerted cytotoxicity in AGS, SGC7901 and MGC803 cells in a dose-dependent manner; *P< 0.05 compared with untreated group. (**b**) AQP3 expression was upregulated in AGS cells and downregulated in MGC803 and SGC7901 cells after lentiviral transduction. (**c**) AQP3 overexpression induced resistance to cDDP compared with the null control in AGS cells, and AQP3 knockdown increased chemosensitivity in MGC803 and SGC7901 cells; *P<0.05. (**d**) AQP3 expression in MGC803, SGC7901 and AGS cells was enhanced after cDDP treatment. UNTR, untreated group.

cDDP is one of the most commonly used chemotherapy drugs for GC. Cell Counting Kit-8 (CCK-8) assays demonstrated that 24 h of cDDP treatment inhibited cell viability of MGC803, SGC7901 and AGS cells in a dose-dependent manner (Figure 1a). The IC_50_ of cDDP was 23.30±1.89, 9.08±0.85 and 26.31±2.07 *μ*M in MGC803, SGC7901 and AGS cells, respectively. A 25, 10 and 30 *μ*M dose of cDDP was used for MGC803, SGC7901 and AGS cells, respectively, in the subsequent experiments. Furthermore, cDDP at these different doses showed little effects on autophagic flux of these GC cells respectively (Supplementary Figure S1). To investigate the effects of AQP3 on chemosensitivity in GC cells, its expression was upregulated in AGS cells and downregulated in MGC803 and SGC7901 cells by lentiviral transduction, as previously reported (Figure 1b).^12,13^ AQP3 overexpression induced cDDP resistance compared with control AGS cells (*P*<0.05, Figure 1c), and AQP3 knockdown increased the chemosensitivity of MGC803 and SGC7901 cells (*P*<0.05, Figure 1c). Furthermore, AQP3 expression in MGC803, SGC7901 and AGS cells was significantly enhanced after cDDP treatment respectively (Figure 1d). Together, these results indicated AQP3 expression modulates the chemosensitivity of GC cells to cDDP.

### AQP3 enhances autophagy in GC cells

**Figure 2:**
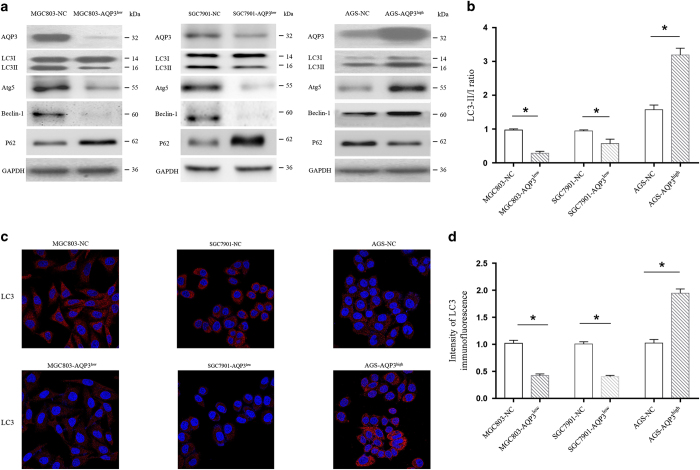
Aquaporin 3 (AQP3) enhances autophagy in gastric cancer cells. (**a**) AQP3 overexpression induced the conversion of LC3-I to LC3-II in AGS cells, and AQP3 knockdown inhibited this conversion in MGC803 and SGC7901 cells (**b**, *P<0.05). AQP3 upregulation increased the expression of Atg5 and Beclin-1, and inhibited P62 expression in AGS cells, whereas AQP3 knockdown produced the opposite results in MGC803 and SGC7901 cells. (**c**) LC3 immunostaining showed that a larger number of small clusters and intensely stained granules were distributed in the cytoplasm near the nuclei of AGS-AQP3^high^ cells compared with AGS-NC cells. However, a certain number of granules were detected in MGC803-NC and SGC7901-NC cells, whereas MGC803-AQP3^low^ and SGC7901-AQP3^low^ cells showed fewer granules (**d**, *P<0.05). Original magnification ×200.

Autophagy has been demonstrated to participate in chemoresistance in GC, but whether AQP3 regulates autophagy to mediate this chemoresistance is unknown. LC3 is one of the most important autophagy-related proteins,^15^ and western blotting demonstrated that AQP3 overexpression promoted the conversion of LC3-I to LC3-II in AGS cells, and AQP3 knockdown inhibited this conversion in MGC803 and SGC7901 cells, suggesting that AQP3 upregulation enhanced autophagy (Figures 2a and b). LC3 immunostaining showed that a large number of small clusters and intensely stained granules were mainly distributed in the cytoplasm near the nuclei of AGS-AQP3^high^ cells compared with control AGS-NC cells. A small number of granules were detected in MGC803-NC and SGC7901-NC cells; however, MGC803-AQP3^low^ and SGC7901-AQP3^low^ cells exhibited fewer than any other condition (Figures 2c and d). Furthermore, AQP3 upregulation increased the expression of Atg5 and Beclin-1, and inhibited P62 expression in AGS cells, whereas AQP3 knockdown produced the opposite results in MGC803 and SGC7901 cells (Figure 2a). These results demonstrate that AQP3 enhances autophagy in GC.

### Chloroquine increases the sensitivity of GC cells to cDDP and reverses AQP3-induced chemoresistance

**Figure 3:**
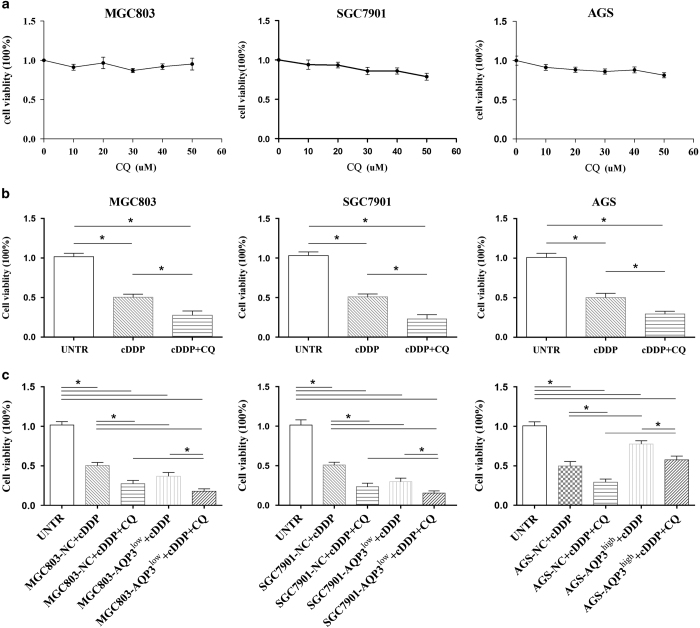
Chloroquine (CQ) increases the sensitivity of gastric cancer cells to cisplatin (cDDP) and reverses the chemoresistance induced by aquaporin 3 (AQP3). (**a**) CQ had little effect on the viability of MGC803, SGC7901 and AGS cells at doses under 50 *μ*M. (**b**) CQ enhanced the effects of cDDP on the proliferation of GC cells in MGC803, SGC7901 and AGS cells; *P<0.05. (**c**) The combination of cDDP and CQ showed more severe cytotoxicity in MGC803-AQP3^low^ and SGC7901-AQP3^low^ cells than in null control (NC) cells or cDDP treatment alone in MGC803-AQP3^low^ and SGC7901-AQP3^low^ cells. The combination therapy significantly reduced the viability of AGS-AQP3^high^ cells compared with cDDP treatment alone. However, the combination therapy exerted less cytotoxicity in AGS-AQP3^high^ cells than in AGS-NC cells, and the effect of combination therapy on AGS-AQP3^high^ cells was similar to cDDP alone on AGS-AQP3^high^ cells; *P<0.05. UNTR, untreated group.

Chloroquine (CQ) has been used to treat malaria; however, it is also regarded as one of the best inhibitors of autophagy.^16^ We used the CCK-8 assay to explore whether CQ could regulate the sensitivity of GC cells to cDDP. Different doses of CQ were first used to test its effect on GC cell viability. CQ had little influence on the viability of MGC803, SGC7901 and AGS cells under a dose of 50 *μ*M (*P*>0.05, Figure 3a); a 20 *μ*M CQ dose was used for further experiments. As a chemosensitizer, CQ enhanced the inhibitory effect of cDDP on GC cell proliferation in MGC803, SGC7901 and AGS cells (*P*<0.05, Figure 3b).

To investigate the role of CQ in AQP3-induced cDDP chemoresistance in GC cells, AGS-AQP3^high^, MGC803-AQP3^low^ and SGC7901-AQP3^low^ cells were treated with a combination of CQ and cDDP. As shown in Figure 3c, the viability of GC cells with silent AQP3 (MGC803-AQP3^low^, SGC7901-AQP3^low^) was significantly reduced compared with the corresponding null control cells (MGC803-NC, SGC7901-NC) treated with cDDP and CQ, or GC cells with silent AQP3 treated with cDDP alone (*P*<0.05, both). The viability of AGS-AQP3^high^ cells was also significantly reduced compared with AGS-AQP3^high^ cells treated with cDDP alone (*P*<0.05, Figure 3c); however, it was superior to that of AGS-NC cells treated with cDDP and CQ (*P*<0.05, Figure 3c); the combination therapy exerted similar cytotoxicity for AGS-AQP3^high^ cells as cDDP alone for AGS-NC (*P*>0.05, Figure 3c). These results indicated that CQ could, at least partly, reverse the chemoresistance to cDDP caused by AQP3 overexpression that may result from the inhibition of AQP3-induced autophagy by CQ.

## Discussion

Recently, AQP3 has drawn the most research interest among all aquaporins.^10,17^ Apart from its expected physiological functions, including urine concentration and exocrine gland secretion, as well as several unanticipated functions, including brain swelling, neural signal transduction, skin moisturization and fat metabolism, it has been implicated in tumorigenesis and tumor progression.^10,18^ AQP3 has been found to be overexpressed in a wide variety of cancer types, and its overexpression has been demonstrated to be associated with tumor invasion, metastasis and poor survival.^19–21^ Our previous studies have established AQP3 as a critical determinant of tumor growth and spread for GC.^7,11–13,22^

cDDP-based therapy remains the most common regimen for GC treatment.^2^ However, cDDP treatment often results in the development of chemoresistance leading to relapse and therapeutic failure. Whether AQP3 participates in chemoresistance of GC remains unclear. Gao *et al.*^14^ have shown that AQP3 and AQP9 overexpression inhibits the therapeutic effect of arsenite in melanoma.^14^ In this study, we showed that AQP3 overexpression is associated with cDDP resistance in GC cells. The artificial upregulation of AQP3 in AGS cells resulted in resistance to cDDP, whereas AQP3 silencing in MGC803 and SGC7901 cells enhanced the cytotoxicity of cDDP. cDDP treatment also significantly upregulated AQP3 expression in GC cells. These results demonstrate that AQP3 mediates chemoresistance to cDDP in gastric cancer cells, consistent with the association between AQP3 overexpression and poor prognosis in GC,^12^ despite the current lack of clinical evidence.

The mechanism underlying AQP3-induced cDDP resistance may be complicated. Gao *et al.*^14^ showed that AQP3 inhibits the therapeutic effect of arsenite in melanoma by upregulating expression of the anti-apoptotic genes *Bcl-2* and *XIAP*, while concurrently downregulating expression of the pro-apoptotic genes *P53* and *BAX*. We demonstrated in this study that AQP3 enhances autophagy in gastric cancer cells. Increased LC3-II levels are commonly used to detect the induction of autophagy *in vitro* and *in vivo*.^23^ AQP3 overexpression induced the conversion of LC3-I to LC3-II in AGS cells, whereas AQP3 knockdown converted LC3-II to LC3-I in MGC803 and SGC7901 cells. Autophagy is a highly regulated catabolic process involving lysosomal degradation of intracellular components, damaged organelles, misfolded proteins and toxic aggregates that reduces oxidative stress and protects cells from damage.^24^ The regulation of autophagy is an evolutionarily conserved and highly complex process, consisting of several basic phases, including initiation, nucleation, maturation and merging with lysosomes, resulting in the degradation of sequestered material.^25^ The process is also induced in response to various conditions, including nutrient deprivation, metabolic stress, hypoxia, anticancer therapeutics and radiation therapy to adapt cellular conditions for survival.^23^ Autophagy can function as a tumor suppressor mechanism in normal cells and dysregulation of this process (that is monoallelic Beclin-1 deletion) may lead to malignant transformation and carcinogenesis. In this study, AQP3 expression was also found to lead to Atg5 and Beclin-1 upregulation, and downregulation of P62. In tumors, autophagy is thought to promote tumor growth and progression by helping cells adapt to and survive in metabolically challenged harsh tumor microenvironments.^24^ Recently, autophagy has been demonstrated to facilitate chemoresistance in a variety of cancers.^26,27^ Therefore, we speculated that AQP3-induced autophagy is associated with cDDP resistance in GC cells.

*In vitro* and *in vivo* studies have demonstrated that pharmacological inhibition (by CQ, hydroxychloroquine or 3-methyladenine) and genetic knockdown of autophagy genes augment the efficacy of various chemotherapeutics and targeted therapies. These studies led to the hypothesis that suppression of the autophagy pathway could be used as a sensitizing strategy for anticancer therapeutics.^28^ CQ (*N*′-(7-chloroquinolin-4-yl)-*N*,*N*-diethyl-pentane-1,4-diamine) is a widely used, safe and effective antimalarial and antirheumatoid agent that has recently been studied for its potential use as an agent that can enhance other cancer therapies.^29^ The lysosomotropic properties of CQ appear to be important for the increase in efficacy and specificity of the combined agent. The early results from several clinical trials indicate that CQ or hydroxychloroquine in combination with anticancer therapies seem to be safe and can augment the efficacy of the various combined therapies.^30,31^ As expected, our data showed that CQ sensitized both GC cells to cDDP. In MGC803 and SGC7901 cells, cell growth was also dramatically suppressed by the combination of CQ and cDDP, greater than cDDP alone when AQP3 was silenced. The cytotoxic effects of the combined therapy was also stronger in AQP3-silenced cells than wild-type cells. In AGS cells, the combination exerted similar effects when AQP3 was upregulated, but the cytotoxic effect of the combination therapy was weaker in cells with AQP3 overexpression than that in wild-type cells. Intriguingly, the combination therapy exerted stronger cytotoxicity in the wild-type AGS cells than in the AGS cells with AQP3 overexpression, and there was no distinction in cell viability between the AGS cells with AQP3 overexpression treated with the combination and the wild-type AGS cells treated with cDDP alone. These results indicated that AQP3 overexpression induced cDDP resistance and CQ reversed this effect, verifying the hypothesis that AQP3 mediates cDDP resistance in GC cells *via* autophagy.

In conclusion, our data show that AQP3 facilitates chemoresistance in GC cells to cDDP, suggesting that the development of AQP3-based tumor therapeutics could enhance the efficacy of cDDP in GC.^10^ Further studies are needed to investigate the molecular details of AQP3-induced autophagy and the other potential mechanisms underlying AQP3-induced chemoresistance.

## Materials and Methods

### Cells and regents

The human gastric carcinoma cell lines, MGC803, SGC7901 and AGS (CBTCCCAS, Shanghai, China), were cultured in RMPI-1640 (Life Technologies, Gibco BRL, Grand Island, NY, USA) supplemented with 10% fetal bovine serum (FBS; Invitrogen, Carlsbad, CA, USA), penicillin/streptomycin (1 : 100 dilution; Sigma, St. Louis, MO, USA) and 4 mM glutamine (Life Technologies) in a humidified atmosphere that contained 5% CO_2_ at 37 °C. cDDP and CQ were purchased from Sigma.

### RNA interference (RNAi)

For small hairpin RNA (shRNA)-mediated AQP3 silencing, a AQP3-RNA interference (RNAi) lentiviral vector was constructed (Shanghai GeneChem Co., Ltd, Shanghai, China). Three candidates of human AQP3-shRNA were synthesized, and the target sequence was 5′- AACGAGGAAGAGAATGTGA-3′ (KD1), 5′- ATGGCTTCTTTGACCAGTT-3′ (KD2) and 5′- GTGGTTTCCTCACCATCAA-3′ (KD3), respectively. These shRNAs were transfected into SGC7901 cells by Lipofectamine 2000 (Invitrogen). As shown in Supplementary Figure S2, KD1 was selected for lentivirus construction. Double-stranded oligonucleotides encoding human AQP3-shRNA (NM_004925) was annealed and inserted into the shRNA expression vector pGC-LV-green fluorescent protein (GFP). The pGC-LV-GFP and pHelper 1.0 and pHelper 2.0 were cotransfected into 293T cells with Lipofectamine 2000. The culture supernatants were collected, concentrated and used as a virus stock. All lentiviral vectors expressed GFP, allowing us to titer and measure their infection efficiency in transfected cells. The viral titer was determined by counting GFP-positive cells. The viral was determined by counting GFP-positive cells after transfection. The lentivirus was applied to transfect MGC803 and SGC7901 cells with a multiplicity of infection (MOI) of 10, and their expression of AQP3 was determined by western blotting (Figure 1b).

### Cell proliferation assays

Cell proliferation was analyzed using the CCK-8 assay (Sigma) according to the manufacturer’s protocol. The results are plotted as mean±S.E. of three independent experiments for each experimental condition.

### LC3-II immunofluorescence

Immunofluorescence was conducted according to the methods reported by Zhou *et al.*^13^ A primary antibody against LC3 A/B (Cell Signaling Technology, Beverly, MA, USA) was used. The secondary antibody was obtained from Beyotime (Beyotime Institute of Biotechnology, Henan, China). Samples were imaged with a 63× objective lens using a ZEISS LSM 710 confocal microscope (Carl Zeiss, Oberkochen, Germany).

### Western blotting

Western blotting was performed to analyze the expression of proteins according to our published methods.^12,13^ We used antibodies against AQP3 (Santa Cruz Biotechnology, Santa Cruz, CA, USA), LC3 A/B (Cell Signaling Technology) and glyceraldehyde-3-phosphate dehydrogenase (GAPDH) (Beyotime Institute of Biotechnology). Densitometric analysis of immunoreactive bands was conducted and results were normalized against GAPDH.

### Statistical analysis

Data are expressed as mean±S.E. In the experiments involving protein expression, the values are representative of three independent experiments. Statistical analysis of the data between the control and treatment groups was performed using analysis of variance. Statistical analyses were performed with SPSS version 17.0 (SPSS Inc., Chicago, IL, USA), and *P*<0.05 was considered statistically significant.
