# Supplementary figures and images for: Aquaporin 3 facilitates chemoresistance in gastric cancer cells to cisplatin via autophagy
Source: Cell Death Discov. 2016 Nov 14;2:16087–. doi: 10.1038/cddiscovery.2016.87 (PMC5107998; doi:10.1038/cddiscovery.2016.87)

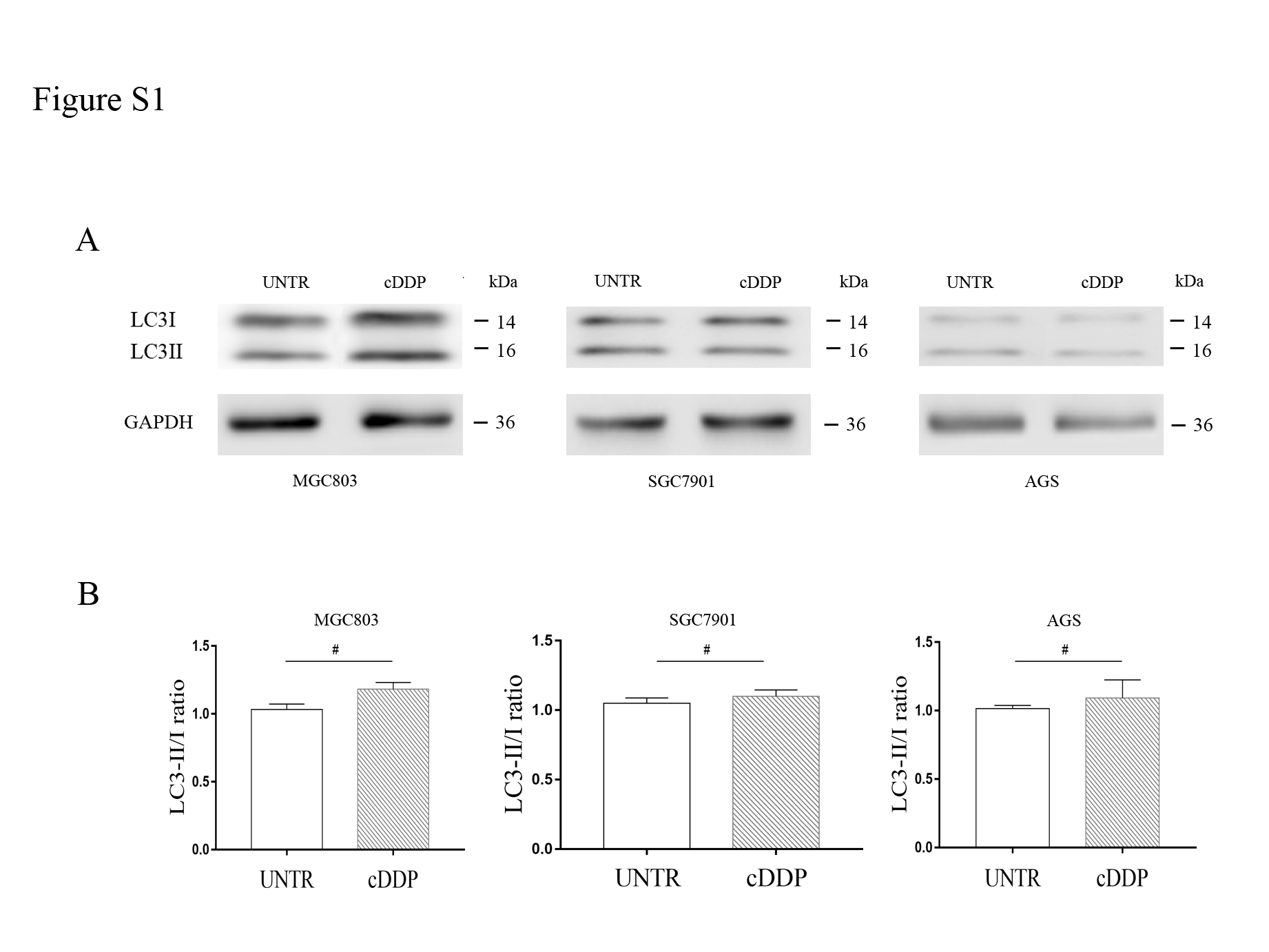

Supplement: Supplementary Figure S1 [file cddiscovery201687-s1.tiff]

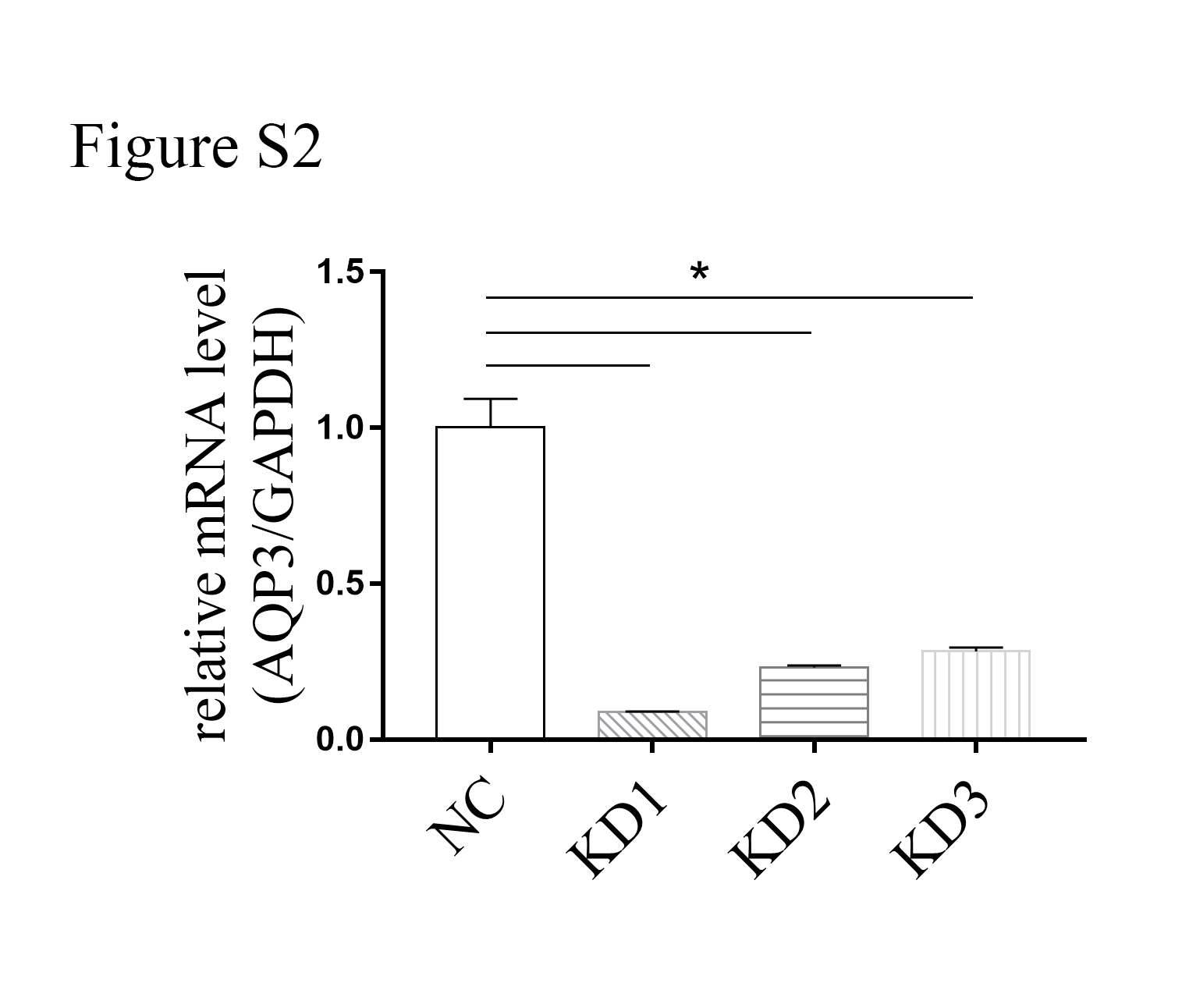

Supplement: Supplementary Figure S2 [file cddiscovery201687-s2.tiff]
