# Supplementary material for: Aquaporin 3 facilitates chemoresistance in gastric cancer cells to cisplatin via autophagy
Source: Cell Death Discov. 2016 Nov 14;2:16087–. doi: 10.1038/cddiscovery.2016.87 (PMC5107998; doi:10.1038/cddiscovery.2016.87)
Supplement: Supplementary Figure Legends [file cddiscovery201687-s3.doc]

**Figure S1.** Cisplatin has no effects on the autophagic flux of MGC 803 (at dose of 25 µM), SGC7901 cells (at dose of 10 µM) and AGS cells (at dose of 30 µM), ^#^*p*＞0.05 compared with the untreated cells (UNTR).

**Figure S2.** Three candidates of human AQP3-shRNA, KD1, KD2 and KD3, exhibits inhibitory effects on AQP3 mRNA, **p*< 0.05 compared with the null control (NC).
